# Pembrolizumab Induced Ocular Hypotony With Near Complete Vision Loss, Interstitial Pulmonary Fibrosis and Arthritis

**DOI:** 10.3389/fonc.2019.00944

**Published:** 2019-09-23

**Authors:** Mike Nguyen, Md Rafiqul Islam, Shueh Wen Lim, Arvind Sahu, Babak Tamjid

**Affiliations:** ^1^Goulburn Valley Health, Shepparton, VIC, Australia; ^2^Royal Victorian Eye and Ear Hospital, Melbourne, VIC, Australia; ^3^Goulburn Valley Health, Peninsula Health, Monash University, Melbourne, VIC, Australia

**Keywords:** melanoma, immunotherapy, immune checkpoint inhibitor, pembrolizumab, immune related adverse event, hypotony, pulmonary fibrosis, arthritis

## Abstract

Clinical outcomes for patients with advanced melanoma have improved significantly with the introduction of immune checkpoint inhibitors. These agents have distinct adverse effects with the potential for heightened host immune responses manifesting as an autoimmune reaction in any organ. We report a unique case who developed pembrolizumab induced arthritis, ocular hypotony with vision loss and pulmonary interstitial fibrosis. A 57-year old gentleman with advanced melanoma was treated with pembrolizumab and attained complete response with no evidence of disease on functional imaging. Treatment was well-tolerated with the only side effect being arthritis controlled with low dose steroids. Following a work related blunt trauma to the right eye, the patient developed bilateral visual impairment secondary to ocular hypotony. The ocular hypotony failed to respond to high-dose glucocorticoid and multiple surgeries. Intraoperatively, ciliary body atrophy was found. Pembrolizumab was ceased after the eye trauma and he remained in complete remission from melanoma. After a further 10 months, the patient developed symptomatic pulmonary fibrosis. There was moderate symptomatic improvement with nintedanib, an antifibrotic agent. This case describes two rare and unique adverse effects. Ocular adverse effects are extremely uncommon and this is the first case to report immune checkpoint inhibitor related ocular hypotony without uveitis to the best of our knowledge. Similarly, the incidence of severe pneumonitis is reported to be low, however limited data is available regarding pulmonary interstitial fibrosis. The occurrence of multiple adverse effects in this case including one occurring several months after cessation of treatment highlights the need for vigilance by clinicians who manage patients treated with immune checkpoint inhibitors. Further research is necessary with regards to rare adverse effects of immune checkpoint inhibitors and the relation of these to treatment administration.

## Introduction

Modern medical oncology practice has been revolutionized with the introduction of immunotherapy, in particular the immune checkpoint inhibitors. These treatments have become increasingly and widely used with efficacy demonstrated in multiple tumor types including melanoma (1–3), non-small cell lung cancer (NSCLC) (4–10), head and neck squamous cell carcinoma (11, 12) and renal cell carcinoma (RCC) (13).

Prior to the introduction of immunotherapy, there were limited treatment options for advanced melanoma and this condition was associated with poor clinical outcomes with median overall survival of <1 year (14). Current immunotherapy treatments widely available in advanced melanoma include monoclonal antibodies against programmed cell death receptor 1 (PD-1) and cytotoxic T-lymphocyte antigen 4 (CTLA-4). Pembrolizumab and nivolumab inhibit PD-1 which is involved in T cell interaction with tumor cells and ipilimumab inhibits CTLA-4 which is involved in the interaction between activated T cells and antigen presenting cells. Tumor induced activation of these receptors dampen the host immune response. The action of these immune checkpoint inhibitors thereby enhances the immune system's anti-tumor effect.

Pembrolizumab has demonstrated efficacy in advanced melanoma with survival rates of 55% and disease control rates of 52% at 24 months in the first line setting (15). Similarly, trials have demonstrated overall survival rates at 3 years of 52% for nivolumab monotherapy and 58% for combination therapy with nivolumab and ipilimumab in first line treatment of advanced melanoma (3). Overall, immune checkpoint inhibitors used as single agents or in combination have improved clinical results with regards to overall survival, progression free survival, best clinical response and can induce a long term durable response.

Immune checkpoint inhibitors have a distinct adverse effect profile which differs from cytotoxic chemotherapy agents. The underlying mechanism for adverse effects is a heightened immune reaction against host tissues which can manifest as an autoimmune process in any organ. Treatment related adverse events are most frequent when immune checkpoint inhibitors are used in combination as observed in the Checkmate 067 trial (3). In this trial, adverse effects of any grade for patients receiving combination therapy occurred in 96% with the most common adverse events being diarrhea, fatigue, pruritus, rash, and nausea. Immune related adverse events (IrAEs) unique to immune checkpoint inhibitors include elevated liver enzymes which occurs in 35% of patients, hyperthyroidism or hypothyroidism 28%, arthralgia 14%, colitis 13%, pneumonitis 7%, and hypophysitis 7%. Ocular side effects are reported to be in <1% of treated patients (16). Most IrAEs respond well when managed with glucocorticosteroids though refractory cases may require additional immunosuppression with steroid sparing agents such as infliximab or mycophenolate (17). We present a unique case of a patient with advanced melanoma who attained complete response with pembrolizumab but developed serious and rare adverse effects.

## Case Presentation

We report a case of a 57 year old male with metastatic melanoma who developed multiple severe immune related adverse effects attributed to pembrolizumab including arthritis, ocular hypotony and pulmonary fibrosis. Four years ago, he was diagnosed with cutaneous melanoma of the upper back measuring 4 mm in thickness associated with ulceration and two positive sentinel lymph nodes. The pathological staging was pT4bN2a. This was treated with wide local excision and regional lymph node clearance. The patient's medical history includes deep vein thrombosis and depression. He is a non-smoker. He did not receive adjuvant treatment and was monitored clinically.

Two years later, he developed metastatic recurrence with cutaneous nodules of melanoma and Positron Emission Tomography (PET) scan demonstrated fluorodeoxyglucose (FDG) avid intrathoracic and intraabdominal lymphadenopathy. Archival tissue analysis did not demonstrate mutations in BRAF gene. He was treated with pembrolizumab (200 mg IV every 3 weeks) and attained complete metabolic response on subsequent PET scans after 3 months of treatment. He continued to receive maintenance pembrolizumab for a total of 32 doses until cessation. After the first 3 months of treatment, he developed arthritis predominantly affecting the small joints of the hands bilaterally, which was associated with mild functional impairment. However, this responded well to low dose glucocorticoids (prednisolone 5 mg daily). The patient was also noted to have developed grade 1 asymptomatic pneumonitis findings on chest CT imaging after 12 months of treatment. These findings were not present at baseline and monitored on serial scans.

After 20 months of treatment and 2 weeks after his last dose, the patient developed reduced vision in the right eye with visual acuity down to <20/200. This was preceded by a work-related blunt trauma injury to his right eye 1 week prior. He was referred to a tertiary eye hospital and exploratory surgery performed excluded a globe rupture. Ophthalmological examination revealed profound ocular hypotony (0 mmHg in right eye) with minimal inflammation. His left vision remained unaffected at this time. Multiple tests and ophthalmological procedures were performed to reverse the hypotony (these will be detailed in a separate ophthalmological report); however, the effect was minimal. Pembrolizumab was ceased thereafter.

Two weeks later, the vision in his left eye started to deteriorate to a visual acuity of only hand movements on the right and counting fingers on the left. Ophthalmological examination confirmed bilateral profound hypotony of 1 mmHg in both eyes, along with fundus changes consistent with hypotony but there was still minimal ocular inflammation. Repeat PET scan excluded recurrence of metastatic disease. He received high dose intravenous and oral steroids and further intraocular surgeries to re-pressurize the eyes, however the response was modest with minimal improvement in vision. It was found intraoperatively that the ciliary processes were pale and atrophic. Despite best efforts, the best attained visual acuity was hand movements in the right eye and 20/120 in the left eye.

Ten months after the cessation of pembrolizumab, the patient developed respiratory symptoms of dyspnoea on exertion, dry cough and progressively worsening exercise tolerance. This was on the background of low-grade inflammatory changes consistent with mild pneumonitis on his prior imaging. High resolution CT chest demonstrated volume loss, asymmetrical interstitial lung markings with honeycomb pattern greater on the left and in predominant subpleural distribution consistent with pulmonary fibrosis. Transthoracic echocardiogram demonstrated normal left ventricular function and moderate pulmonary hypertension (RVSP 47 mmHg). Pulmonary function test demonstrated moderately severe restrictive ventilatory defect and reduced diffusion capacity of lung for CO of 49% predicted. Six-minute walk test demonstrated exertional hypoxia corrected with supplemental oxygen. Bronchoscopy did not yield any infective pathogens. There were no laboratory findings suggestive of connective tissue disorders. A course of oral glucocorticoids provided limited effect. The antifibrotic agent, nintedanib was commenced but resulted in only moderate symptomatic improvement in respiratory symptoms and caused gastrointestinal toxicity with diarrhea despite dose reductions. At the time of this report, the patient remains in complete remission from melanoma, 18 months after treatment cessation. However, he remains severely affected by the adverse effects of severe visual impairment and progressive pulmonary fibrosis.

## Discussion

There is an increasing number of patients being treated with immune checkpoint inhibitors, thus clinicians should be well aware of the potential immune-related adverse effects as prompt recognition and management is critical. Common adverse effects of PD-1 inhibitors occurring in more than 10% of patients include fatigue, rash, diarrhea, cough, pruritus, arthralgia, nausea, and hypothyroidism. Less frequent but potentially severe and fatal immune related adverse events include pneumonitis, severe colitis, hepatitis, hypophysitis, diabetes mellitus, and nephritis [17]. CTLA-4 antibodies can cause more frequent and higher grade adverse effects. IrAEs have a classic pattern whereby rash and pruritus begin within 3 weeks of treatment, gastrointestinal side effects such as colitis begin within 10 weeks and hepatitis and endocrine side effects appear later (17). However, IrAEs can occur at any time, even after the cessation of immune checkpoint inhibitors.

Ocular side effects are rare, occurring in <1% of patients. Any component of the ocular apparatus can be affected. The most common side effects are dry eyes, conjunctivitis, uveitis, and myasthenia gravis. Cases of keratitis, thyroid like orbitopathy, optic neuritis, retinal vasculitis, and choroiditis have been described (16). It is important to exclude metastatic disease and paraneoplastic syndrome as potential mimics. Hypotony is the presence of low intraocular pressure defined as below 6 mmHg (18), and commonly presents with decreased vision. Uveitis can cause hypotony as chronic inflammation can result in atrophy of the ciliary epithelium, leading to reduced production of aqueous humor (19). Uveitis-related hypotony is managed with high dose local or systemic corticosteroids and is usually highly responsive.

Pembrolizumab-induced uveitis with or without hypotony has been reported in the literature. Basilious & Lloyd reported a case of pembrolizumab-induced uveitis, hypotony and cataracts with minimal improvement despite cessation of pembrolizumab, prolonged corticosteroid treatment and multiple ophthalmic procedures (20). In contrast, Samra et al. reported a case of pembrolizumab-induced uveitis and papillitis which resolved quickly and completely with treatment cessation and topical steroids (21). Hanna reported a case of pembrolizumab-induced panuveitis with choroiditis which resolved after a prolonged course of systemic steroids (22). A systematic review found 10 published cases of uveitis related to immune checkpoint inhibitors and report an odds ratio of 3.4 for any ocular toxicity compared with non-immune checkpoint inhibitor therapies (23).

Our case is distinct in that this patient developed bilateral ocular hypotony in the absence of uveitis. In addition, he had a history of trauma to one eye which resulted in visual impairment, but subsequently developed visual impairment in the contralateral eye several weeks later. Trauma to one eye may have exposed the immune system to antigens usually concealed within the ophthalmic apparatus, and anti-PD1 therapy may have accelerated the autoimmune activity to the contralateral eye. This was the proposed theory, however, if this was the only mechanism, we would expect more significant uveitis and a greater response to the high dose corticosteroids given.

Pulmonary adverse effects to immune checkpoint inhibitors are extensively reported and range from non-specific dyspnoea, infective pneumonia, inflammatory pneumonitis, organizing pneumonia and interstitial pulmonary fibrosis. Checkpoint inhibitor associated pneumonitis predominates in the published literature with fewer reports related to interstitial fibrosis. A meta-analysis by Nishino et al. found the overall incidence of pneumonitis of 2.7% with a higher incidence in NSCLC and RCC compared with melanoma; and pneumonitis occurring more frequently with combination immune checkpoint inhibitor therapy compared with monotherapy (24). Delaunay et al. (25) published a case series of immune checkpoint inhibitor associated interstitial lung disease (ILD), incorporating pneumonitis and fibrosis, with overall incidence of 3.5%. The majority of cases occurred in patients with NSCLC (75%) followed by melanoma (20%). ILD typically occurs during the first months of treatment (26, 27), in contrast to this case where it occurred 30 months from the start of treatment.

This case highlights the potential for the development of multiple IrAEs with immune checkpoint inhibitors. Clinical trials of immune checkpoint inhibitors commonly report adverse effects as a composite figure of any grade of event, thus limiting the full appreciation of patients who develop multiple concurrent or sequential adverse effects. Notwithstanding this, there are several case reports of patients experiencing multiple severe adverse effects to immune checkpoint inhibitors (26). Sbeih et al. (27) reported a single center experience with 18% of patients experiencing multiple IrAEs and this was associated with better survival. A separate multi-center retrospective analysis reported 13% of patients had more than one concurrent IrAEs whilst receiving combination immune checkpoint therapy and 21% of patients developed another distinct IrAE upon resumption of immune checkpoint therapy (28). This case is unique in that arthritis, pneumonitis and ocular hypotony occurred sequentially during treatment, and pneumonitis progressed to symptomatic pulmonary fibrosis several months after the cessation of therapy.

Our patient attained a complete response with no detectable melanoma whilst on treatment with pembrolizumab and maintained this response despite cessation of treatment, thus indicating an excellent long term prognosis. In patients who attain complete response, the risk of developing progressive disease is low with disease-free survival at 24 months of over 90%. Similarly, there is low risk of disease progression after pembrolizumab therapy is ceased with disease free survival of almost 86% 24 months after cessation of pembrolizumab (29).

In conclusion, the use of immune checkpoint inhibitors in medical oncology continues to rise significantly given the dramatic improvements in clinical outcomes across a range of tumor types. These treatments have the potential for a wide range of systemic adverse effects, particularly IrAEs which can affect any part of the body. This is the first published case of a patient with pembrolizumab-induced arthritis, bilateral ocular hypotony with severe vision loss and pulmonary fibrosis requiring treatment with anti-fibrotic agents. The unique composition of the individual adverse effects and relation to the timing of treatment in this case highlight the need for clinical vigilance of rare but severe immunotherapy-related side effects. Further research is required regarding the optimal duration of immune checkpoint inhibitor therapy, especially in patient who achieve complete response.

## Ethics Statement

Ethical review and approval was not required for the study on human participants in accordance with the local legislation and institutional requirements. The patients/participants provided their written informed consent to participate in this study. Written informed consent was obtained from the individual(s) for the publication of any potentially identifiable images or data included in this article.

## Author Contributions

All authors listed have made a substantial, direct and intellectual contribution to the work, and approved it for publication.

### Conflict of Interest

The authors declare that the research was conducted in the absence of any commercial or financial relationships that could be construed as a potential conflict of interest.

## Abbreviations

NSCLC: non-small cell lung cancer
RCC: renal cell carcinoma
PD-1: programmed cell death receptor 1
CTLA-4: cytotoxic T-lymphocyte antigen 4
IrAEs: Immune related adverse events
FDG: fluorodeoxyglucose
ILD: interstitial lung disease.
